# *Salmonella enterica* relies on carbon metabolism to adapt to agricultural environments

**DOI:** 10.3389/fmicb.2023.1213016

**Published:** 2023-09-07

**Authors:** Min Han, Jasper Schierstaedt, Yongming Duan, Monika Nietschke, Sven Jechalke, Jacqueline Wolf, Michael Hensel, Meina Neumann-Schaal, Adam Schikora

**Affiliations:** ^1^Federal Research Centre for Cultivated Plants, Julius Kühn Institute (JKI), Institute for Epidemiology and Pathogen Diagnostics, Braunschweig, Germany; ^2^Department Plant-Microbe Systems, Leibniz Institute of Vegetable and Ornamental Crops (IGZ), Großbeeren, Germany; ^3^Division of Microbiology, Biology/Chemistry, University of Osnabrück, Osnabrück, Germany; ^4^Institute of Phytopathology, Research Centre for Biosystems, Land Use and Nutrition (IFZ), Justus-Liebig-University Gießen, Gießen, Germany; ^5^Leibniz Institute DSMZ-German Collection of Microorganisms and Cell Cultures, Braunschweig, Germany

**Keywords:** *Salmonella*, carbon metabolism, diluvial sand soil, lettuce, tomato, fresh produce

## Abstract

*Salmonella enterica*, a foodborne and human pathogen, is a constant threat to human health. Agricultural environments, for example, soil and plants, can be ecological niches and vectors for *Salmonella* transmission. *Salmonella* persistence in such environments increases the risk for consumers. Therefore, it is necessary to investigate the mechanisms used by *Salmonella* to adapt to agricultural environments. We assessed the adaptation strategy of *S. enterica* serovar Typhimurium strain 14028s to agricultural-relevant situations by analyzing the abundance of intermediates in glycolysis and the tricarboxylic acid pathway in tested environments (diluvial sand soil suspension and leaf-based media from tomato and lettuce), as well as in bacterial cells grown in such conditions. By reanalyzing the transcriptome data of *Salmonella* grown in those environments and using an independent RT-qPCR approach for verification, several genes were identified as important for persistence in root or leaf tissues, including the pyruvate dehydrogenase subunit E1 encoding gene *aceE*. *In vivo* persistence assay in tomato leaves confirmed the crucial role of *aceE*. A mutant in another tomato leaf persistence-related gene, *aceB*, encoding malate synthase A, displayed opposite persistence features. By comparing the metabolites and gene expression of the wild-type strain and its *aceB* mutant, fumarate accumulation was discovered as a potential way to replenish the effects of the *aceB* mutation. Our research interprets the mechanism of *S. enterica* adaptation to agriculture by adapting its carbon metabolism to the carbon sources available in the environment. These insights may assist in the development of strategies aimed at diminishing *Salmonella* persistence in food production systems.

## Introduction

*Salmonella enterica* is an important foodborne pathogen, causing an estimated 1.35 million and 91,000 human cases in the United States and the European Union every year (CDC, 2022b; EFSA, 2022b). Aside from contaminations originating in livestock-based products such as meat, eggs, dairy, and derived cheese or butter, raw-consumed plant produce plays an increasing role in *Salmonella* contaminations (Carrasco et al., 2012). Salad vegetables, such as lettuce (*Lactuca sativa*), tomato (*Solanum lycopersicum*), cabbage (*Brassica oleracea*), and onions (*Allium cepa* L.), have been reported to cause outbreaks in the past years by the CDC (2022a). To diminish the risk of contamination, in addition to quality control of fertilizer sources, irrigation water, and farming activities (Schierstaedt et al., 2019), the persistence of *Salmonella* in plant-related environments should be added to the risk assessment. *Salmonella* has been observed to survive for weeks in soil, seeds, leaves, and blossoms, though the length of persistence varies depending on the situation (Van der Linden et al., 2013; Zheng et al., 2013; Jechalke et al., 2019; Zarkani et al., 2019; Montano et al., 2020; Schierstaedt et al., 2020).

In a typical host environment, such as mammalian cells or tissues, *Salmonella* employs genes encoded on specific genetic regions, known as *Salmonella* Pathogenicity Islands (SPIs), for invasion, reproduction, and colonization. Type III Secretion Systems (T3SSs) and effectors encoded on diverse SPIs assist *Salmonella* in overcoming the host's innate immunity. In atypical hosts, for example, plants, the use of T3SSs and effectors remains ambiguous, and corresponding reports are sometimes conflicting. SPI1 and SPI2 were shown to be implicated in the colonization of Arabidopsis (*Arabidopsis thaliana*) and lettuce (Schikora et al., 2011; Garcia et al., 2014; Neumann C. et al., 2014; Chalupowicz et al., 2018; Johnson et al., 2020; Montano et al., 2020). However, none of the SPI-associated genes was upregulated in response to tomato (Zarkani et al., 2019).

*Salmonella*-specific responses are crucial for successful invasion and colonization. Efficient utilization of available nutrients, microelements, and energy sources plays an important role as well. Carbon, nitrogen, oxygen, and hydrogen occur in diverse chemical compounds in various environments, providing *Salmonella* with components for protein, lipid, carbohydrate, and nucleic acid metabolism. Efficient use of carbon sources in different environments benefits microorganisms, including *Salmonella*, for colonization and persistence in a particular ecological niche (Sit et al., 2015; Cole et al., 2017; Prusky and Wilson, 2018; Zhang et al., 2018; Hudson et al., 2022). The most common carbon source for *Salmonella* is glucose, which is catabolized by central carbon metabolism pathways including glycolysis, pyruvate oxidation, and the tricarboxylic acid (TCA) cycle (Bowden et al., 2009). Glycolysis is the initial step in glucose catabolism, and it is linked to the TCA cycle via pyruvate oxidation. *Salmonella* mutants in genes encoding glycolysis intermediate metabolic enzymes such as *enolase* (*eno*), *fructose-bisphosphate aldolase* (*fba*), *phosphoglycerate kinase* (*pgk*), *glyceraldehyde-3-phosphate dehydrogenase* (*gapA*), and *triosephosphate isomerase* (*tpiA*) are deficient in survival in macrophage cells (Dandekar et al., 2012). In addition, the pentose phosphate pathway (PPP) and the Entner–Doudoroff pathway (KDPGP) contribute to glucose catabolism in *Salmonella*, though not as substantially as glycolysis (Bowden et al., 2009). Furthermore, the glyoxylate pathway is another vital anaplerotic process (Cronan and Laporte, 2005). Apart from C6 carbohydrates, *Salmonella* can use a variety of intermediates as carbon sources, including several C4 dicarboxylates (such as succinate, fumarate, malate, aspartate, and tartrate) (Clark and Cronan, 2005; Unden et al., 2016). Accordingly, metabolic processes in *Salmonella* may be dynamically modified in order to guarantee its survival. In infected mice's gut, *Salmonella* is capable of catabolizing the succinate produced by other gut microorganisms and therefore completing its own TCA cycle (Spiga et al., 2017). To overcome colonization resistance built by gut microbiota, *Salmonella* outperforms the host carbon requirements for lactate (Gillis et al., 2018). The successful utilization of carbon sources allows *Salmonella* to grow in quite different environments. Interestingly, comparisons between *Salmonella* metabolism while present in plants and during intestinal colonization revealed similarities (Kwan et al., 2018).

In this study, we hypothesized that *Salmonella enterica* serovar Typhimurium strain 14028s (*S*. Typhimurium 14028s) actively adapts to agricultural environments as ecological niches and changes its metabolism accordingly. In the first step, GC-MS analysis was applied to assess the availability of intermediate compounds in central carbon metabolism in diverse ecological niches related to agricultural systems, which included diluvial sand (DS) soil suspension, tomato leaf-based medium (TM), and lettuce leaf-based medium (LM). In parallel, the same compounds were assessed in correspondingly cultured *Salmonella* cells. Furthermore, genes advantageous for *Salmonella* grown in root- or leaf-related environments were identified by the comparison of transcriptome data (Jechalke et al., 2019; Zarkani et al., 2019; Schierstaedt et al., 2020) and further verified by reverse transcription quantitative PCR (RT-qPCR). Two genes seemed of particular interest, namely the *pyruvate dehydrogenase subunit E1* (*aceE*) and *malate synthase A* (*aceB*). Mutated and complemented strains in *aceE* and *aceB* were subjected to persistence assay in tomato leaves in order to validate our observations *in vivo*. In addition, major intermediates of central carbon metabolism were compared between *S*. Typhimurium 14028s and the mutants. Our results suggest that fumarate accumulation contributed to the enhanced persistence of the *aceB* mutant. The downregulation of fumarate catabolism genes in the *aceB* mutant, compared to *S*. Typhimurium 14028s, suggests a possible route for fumarate accumulation. Taken together, our research provides new insights into how *Salmonella* modifies its own carbon metabolism in order to thrive in agricultural environments.

## Materials and methods

### Bacterial strains

*Salmonella enterica* serovar Typhimurium strain 14028s (*S*. Typhimurium 14028s) with spontaneous rifampicin resistance (50 μg/mL) used in this study was constructed by Fornefeld et al. (2017). *S*. Typhimurium 14028s single gene mutation strains (Δ*aceA*, Δ*aceB*, and Δ*aceE*) with kanamycin resistance (50 μg/mL) were provided by Michael Hensel (Universität Osnabrück, Germany). Since *aceB* and *aceE* are the first genes in corresponding operons, the complete sequences of *aceBAK* and *aceEF* were cloned and introduced to Δ*aceB* and Δ*aceE*, respectively. Complemented strains (Δ*aceB*::*aceBAK* and Δ*aceE*::*aceEF*) are both kanamycin (50 μg/mL) and carbenicillin (50 μg/mL) resistant. Briefly, sequences were cloned to the backbone of pWSK29. The vectors (PaceB aceBAK and PaceE aceEF) were thereafter transformed into *Escherichia coli* strain NEB5α (New England Biolabs, USA), respectively. Single colonies picked from medium-added carbenicillin (50 μg/mL) were verified by sequencing. Afterward, vectors of PaceB aceBAK and PaceE aceEF extracted from *E.coli* NEB5α were electroporated using the Ec2 program in the MicroPulser Electroporator (Bio-Rad, Germany) into Δ*aceB* and Δ*aceE* competent cells, respectively. After 1 h of recovery in SOC medium [SOB (Carl Roth GmbH + Co. KG, Germany) + 20 mM glucose (Carl Roth GmbH + Co. KG, Germany)] at 37°C with a shaking speed of 150 rpm, the bacterial suspension was spread on LB agar containing kanamycin (50 μg/mL) and carbenicillin (50 μg/mL) and incubated at 37°C overnight. Single colonies were picked, streaked onto novel plates, and verified by PCR. Primers for verification are listed in Supplementary Table S1.

### Medium recipes and culture conditions

*Salmonella* was cultured in LB medium (Carl Roth GmbH + Co. KG, Germany) with the required antibiotics for inoculums. Minimal medium (MM) was prepared from 12.3 mM glucose, 1 × M9 salts (5 × , Sigma-Aldrich Chemie GmbH, Germany), 2 mM MgSO_4_ (Carl Roth GmbH + Co. KG, Germany), and sterile deionized water (Jechalke et al., 2019). Diluvial sand (DS) soil suspension is composed of DS soil (Rühlmann and Ruppel, 2005) and 10 mM MgCl_2_ in a 1:2 (m/m) proportion (Schierstaedt et al., 2020). Tomato and lettuce leaf-based media (TM and LM) contain 25% (v/v) of leaf homogenates, 20% (v/v) M9 salts, and 55% (v/v) sterile deionized water (Fornefeld et al., 2017; Zarkani et al., 2019). Leaf homogenates were prepared from 45 g of blended leaves in 300 mL of sterile deionized water and afterward filtered through two layers of paper tissues. Tomato and lettuce root exudate-based media (TE and LE) were made by mixing root exudates with 10 mM MgCl_2_ (Jechalke et al., 2019; Zarkani et al., 2019). The collection of root exudates was executed as previously published (Witzel et al., 2017). Briefly, plant roots were washed and immersed in sterile deionized water for 1 h, followed by another 4 h of immersion in fresh water. A suspension of every 25 plants was filtered (pore size of 0.22 μm) and concentrated using freeze-drying as one sample. XLD agar (Carl Roth GmbH + Co. KG, Germany) was used for the selective enumeration of *Salmonella*. *Salmonella* cultures were grown at 37°C in LB and on XLD and at 28°C in environment-related media.

### Collection of *Salmonella* samples *in vitro*

A dialysis membrane system was used to collect *Salmonella* samples *in vitro* for metabolite measurement and RT-qPCR assays (Han et al., 2023). In brief, *Salmonella* cultures in the stationary phase were centrifuged, washed, and diluted in 10 mM MgCl_2_ to 10^9^ CFU/mL. Three milliliters of the dilution were added into a cellulose ester dialysis membrane (MWCO 100,000, ϕ = 16 mm, Spectra/Por Biotech, USA) bag, which was then sealed and immersed in 30 mL of the respective medium in a 50 mL centrifuge tube. The tube was shaken at 28°C for 24 h. In the metabolism experiment, part of the samples was separated for bacterial enumeration, while the rest was pelleted at 4°C in a precooled centrifuge tube. After being rinsed on ice with 10 mM MgCl_2_, the pellets were centrifuged again and instantly frozen in liquid nitrogen. *Salmonella* cultures designated for RNA extraction were immediately vortexed with double the volume of RNAprotect Bacteria Reagent (Qiagen, Germany). After 5 min of incubation, the bacteria were centrifuged at 5,000 × *g* for 10 min at room temperature. Bacterial pellets for both metabolism and RT-qPCR assays were stored at −80°C until further processing. Four biological replicates were prepared for each treatment.

### Metabolite extraction, GC-MS, and data analysis

Metabolites were extracted from wet biomass through the methanol–water–chloroform extraction process using 100 μL of methanol/^13^C-ribitol solution to re-suspend the cells. Thereafter, the sequential addition of 100 μL of H_2_O and 150 μL of chloroform was performed with vortexing for 5 min in between. After centrifugation, 150 μL of the polar phase was dried in a vacuum concentrator. For extracellular metabolites, 10 μL of cell-free culture supernatant was mixed with 10 μL of methanol/^13^C-ribitol solution and then dried in a vacuum concentrator. Derivatization and GC-MS measurement (splitless and split 1:10) of metabolites were performed as described in a previous study (Will et al., 2019). Processing of raw data was performed using the MetaboliteDetector (Neumann-Schaal et al., 2015). The peak area data were normalized to the internal standard ^3^C-ribitol and—for intracellular metabolites—to the determined cell number of the harvested cells. Data are available on FAIRDOMHUB (seek ID: https://fairdomhub.org/projects/348). MetaboAnalyst 5.0 (https://www.metaboanalyst.ca/), a web-based tool, was used for analysis. The principle component analysis (PCA) was performed using the statistical analysis (one factor) module. In the heatmaps, the cluster method is Ward.D and the distance is Euclidean distance. The relative abundance of metabolites in *S*. Typhimurium 14028s and Δ*aceB* grown in TM was compared to that in MM using log_2_ fold change. Four biological replicates were prepared for each treatment.

### *Salmonella* RNA extraction, reverse transcription, and RT-qPCR

*Salmonella* total RNA was extracted using the RNeasy Mini Kit (QIAGEN, Germany), DNA was eliminated, and cDNA was synthesized using the Maxima H Minus First Strand cDNA Synthesis Kit (Thermo Fisher Scientific, USA) according to the manufacturer's instructions. Thereafter, 20 μL of the cDNA generated from 1 μg DNA-digested RNA was diluted to 100 μL for qPCR. The qPCR reaction system was 20 μL, consisting of 10 μL of LUNA Master Mix (New England Biolabs, USA), 4 μL of nuclease-free water, 0.5 μL of forward or reverse primers (10 μM), and 5 μL of diluted cDNA. The Bio-Rad CFX Connect cycler (Bio-Rad, Germany) was programmed with an initial heating of 95°C for 5 min, followed by 39 cycles of 95°C for 15 s and 60°C for 30 s (+plate read). Melting curves were measured for quality control. The *rfaH* gene (Cardenal-Munoz and Ramos-Morales, 2013) was used as a calibrator for raw data normalization. The fold change was calculated using the 2^−Δ*ΔCt*^ method (Livak and Schmittgen, 2001) compared to gene expression in MM. Unpaired Student's *t*-test or one-way ANOVA with the Tukey HSD test was used to assess differences. The primers used are listed in Supplementary Table S1. Two technical replicates of the qPCR reaction were set for each sample, and the error bars represented the standard deviation among the four biological replicates.

### Plant cultivation

Three weeks after sowing, tomato (*Solanum lycopersicum* cultivar Moneymaker) seeds germinated in Substrate 1 (Klasmann-Deilmann GmbH, Germany) were transplanted to 9 cm diameter pots in the greenhouse with 18 h of daylight at 20°C. To avoid contamination of the leaves, plants were irrigated and fertilized from the bottom of the trays as needed. Plants were ready for treatment 3 weeks after transplantation.

### Persistence assay

To evaluate the persistence of *S*. Typhimurium 14028s, single gene mutant strains, and complemented strains in tomato leaves, 6-week-old plants cultivated as indicated above were gently infiltrated with bacterial suspension (10^7^ CFU/mL). The most marginal two leaflets of the plant's second and third leaves were chosen. When the inoculated leaflets recovered from their water-stained appearance 3 h post-infiltration, as well as 7 and 14 days post-inoculation (dpi), four 5-mm diameter leaf disks were sampled and pooled in a 2 mL centrifuge tube, homogenized with 200 μL of 10 mM MgCl_2_ using a motor handpiece (Xenox, Germany), and then replenished to 1 mL. Two technical replicates of homogenized leaf slurry were serially diluted in a 96-well plate. Ten μL of the dilution was dropped on XLD agar supplemented with appropriate antibiotics in duplicate. After overnight incubation at 37°C, *Salmonella* colonies with easily identifiable black coloration were enumerated. The persistence was evaluated by calculating the log_10_ CFU in each leaf disk. One-way ANOVA with the Tukey HSD test was used to verify differences. The error bars represented the standard deviation among the four biological replicates.

## Results

### Agricultural environments have different availabilities of carbon compounds that influence *Salmonella* metabolism

*Salmonella enterica* serovar Typhimurium strain 14028s (*S*. Typhimurium 14028s) was reported to persist in diluvial sand (DS) soil for several weeks (Schierstaedt et al., 2020), as well as in tomato and lettuce leaves (Jechalke et al., 2019; Zarkani et al., 2019). Carbon utilization is critical for microbial adaptation to agricultural environments. *Salmonella*'s central carbon metabolism is based on glycolysis, pyruvate oxidation, and the tricarboxylic acid (TCA) cycle. To determine whether *S*. Typhimurium 14028s used a carbon metabolism adaptation strategy to persist in agricultural environments, the abundance of the central carbon metabolism intermediate products was evaluated both in the mimicking media [DS soil suspension, tomato leaf-based media (TM), and lettuce leaf-based media (LM)] to imply the availability for bacteria and in the correspondingly incubated *S*. Typhimurium 14028s cells.

**Figure 1:**
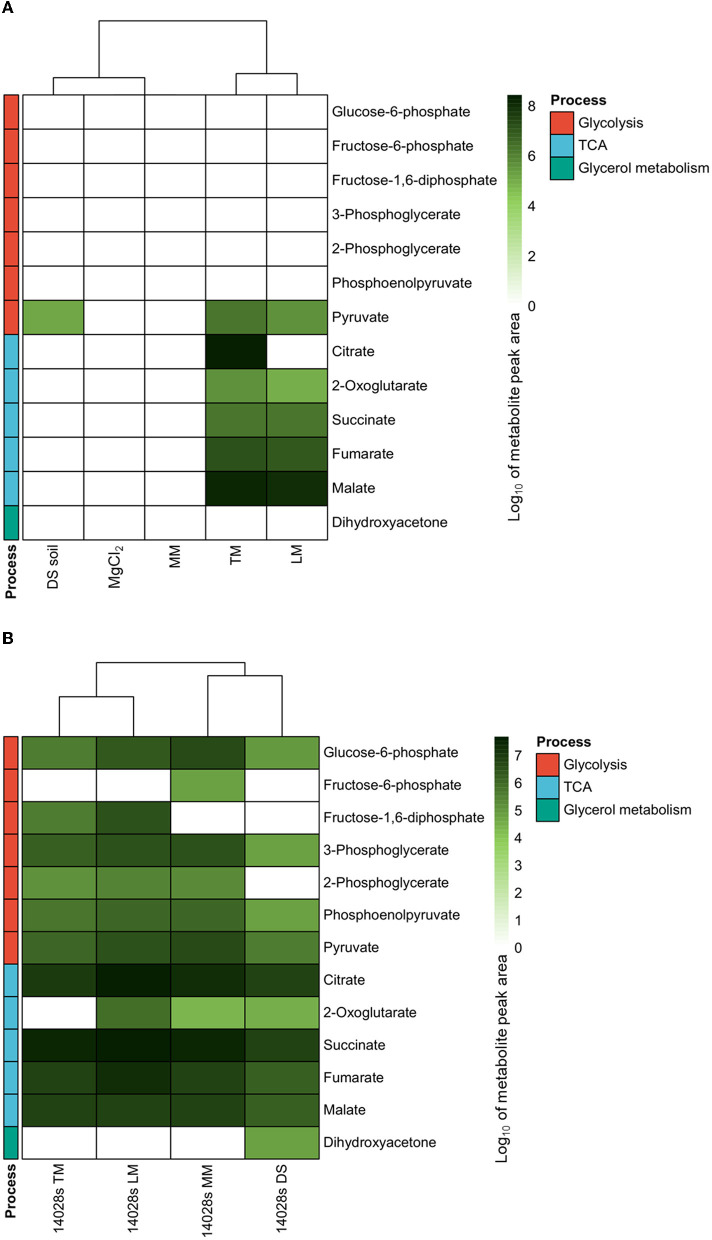
Central carbon metabolism intermediates in environments and correspondingly cultured *Salmonella*. Major intermediates of glycolysis and the tricarboxylic acid (TCA) cycle were measured using GC/MS. The abundances in media **(A)**, including 10 mM MgCl_2_, minimal medium (MM), diluvial sand (DS) soil suspension, tomato leaf-based medium (TM), and lettuce leaf-based medium (LM), as well as correspondingly cultured *Salmonella enterica* serovar Typhimurium strain 14028s (*S*. Typhimurium 14028s) **(B)**, were evaluated. Normalized peak area data from GC/MS were indicated by log_10_ in the heatmap. The cluster method is Ward.D and the distance is Euclidean distance.

As references, 10 mM MgCl_2_ and M9 salt-based minimal medium (MM) were used. MgCl_2_ solution, which was used to pre-suspend the bacteria before inoculation and to wash pellets during sampling, displayed a clean background for all metabolites discussed below (Figure 1A). The MM, a minimal environment provided for *S*. Typhimurium 14028s basal carbon metabolism, did not contain any of the evaluated compounds, except glucose, which was added as the only carbon source and was overloaded in the measurement. Except for pyruvate, DS soil, the sandy soil collected from fields (Rühlmann and Ruppel, 2005), was poor in the evaluated central carbon metabolism intermediate compounds. Glycerol, on the other hand, was of extraordinarily high abundance (overloaded) and therefore a possible carbon source. By contrast, TM and LM were rich in nutrients. Excess glucose and fructose were detected (overloaded) in both media, as well as several di/trisaccharides in LM. Furthermore, pyruvate, a glycolysis end product, and the TCA cycle intermediates, including 2-oxoglutarate, succinate, fumarate, and malate, were also present in TM and LM (Figure 1A).

Differences in the composition of carbon-related metabolites in different environments could influence bacterial metabolism. Therefore, in the next step, related compounds were evaluated in *S*. Typhimurium 14028s. No difference in the cell multiplication of *S*. Typhimurium 14028s grown in TM, LM, or MM was observed at 24 hpi (Supplementary Figure S1). However, *S*. Typhimurium 14028s inoculated into DS soil suspension presented no change in the cell number at the harvest time point compared to the inoculum (Supplementary Figure S1). A clear dispersion was observed between central carbon metabolism intermediates present in bacterial cells under different culturing conditions. In a principal component analysis (PCA), the first PC1 (65.1%) and second PC2 (20%) dimensions explained 85.1% of the data variance and separated the data into treatment-related populations (Supplementary Figure S2). All four biological replicates were included in 95% confidence intervals. Detailed analysis revealed that, in *S*. Typhimurium 14028s introduced to MM, glycolysis intermediates including glucose-6-phosphate, fructose-6-phosphate, 3-phoshoglycerate, 2-phoshoglycerate, phosphoenolpyruvate, and pyruvate were present, although fructose-1,6-diphosphate was not detectable (Figure 1B). The situations in bacterial cells inoculated with TM and LM were slightly different. Instead of fructose-1,6-diphosphate, fructose-6-phosphate was not detectable in *Salmonella* cells in TM or LM. In DS soil, where glycerol was available rather than glucose, neither fructose-6-phospahte nor fructose-1,6-diphopsphate was detectable in *S*. Typhimurium 14028s cells. Furthermore, no 2-phosphoglycerate was detected. Dihydroxyacetone, the direct downstream product of glycerol catabolism, was present and abundant instead, indicating glycerol consumption activity in cells introduced to DS soil. TCA metabolites such as citrate, succinate, fumarate, and malate were detected in all samples. Interestingly, 2-oxoglutarate was not detectable in *S*. Typhimurium 14028s grown in TM (Figure 1B).

The results presented above strongly suggest that the availability of carbon compounds varied in diverse agricultural environment-related media, as well as the metabolite content of *S*. Typhimurium 14028s grown in those conditions (Figure 1). Predicatably, TM and LM media clustered close together, whereas the composition of DS soil suspension was closer to 10 mM MgCl_2_ solution and MM (Figure 1A). Interestingly, the metabolite pattern of *S*. Typhimurium 14028s exhibited a highly comparable clustering pattern (Figure 1B). It was, therefore, reasonable to speculate that *S*. Typhimurium 14028s metabolism is related to the ecological niche, for example, root or leaf.

### Specific genes are regulated in *Salmonella* grown in root- and leaf-related environments

Since the metabolism appeared dependent on a root- or leaf-related ecological niche, we speculated which *Salmonella* genes potentially contributed to the adaptation. The transcriptome of *S*. Typhimurium 14028s incubated in DS soil suspension (Schierstaedt et al., 2020), TM (Zarkani et al., 2019), and LM (Jechalke et al., 2019) for 24 h was indicated in previous studies. Additionally, root exudates are another important component of the root environment. The transcriptome of *S*. Typhimurium 14028s cells incubated in tomato or lettuce root exudate-based media (TE and LE, respectively) provided supplementary information on their transcriptional adjustment to root-related niches (Jechalke et al., 2019; Zarkani et al., 2019).

**Figure 2:**
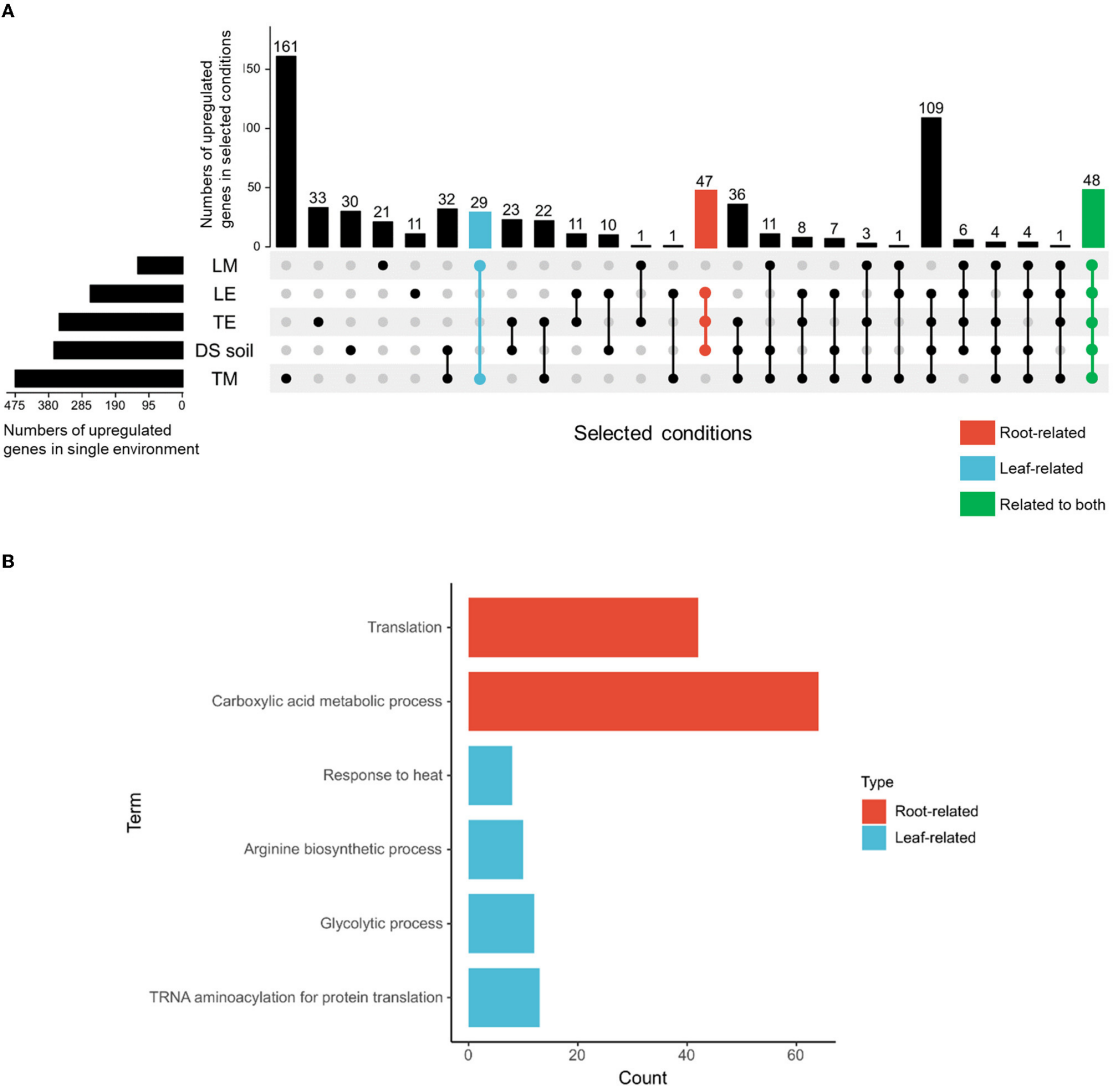
Upregulated genes and enriched GO terms contributing to *S*. Typhimurium 14028s adaptation to root-, leaf-, or related environments. Upregulated genes of *S*. Typhimurium 14028s incubated in DS soil suspension (Schierstaedt et al., 2020), tomato root exudate-based medium (TE) (Zarkani et al., 2019), lettuce root exudate-based medium (LE) (Jechalke et al., 2019), TM (Zarkani et al., 2019), and LM (Jechalke et al., 2019) were extracted and compared. DS soil suspension, TE, and LE were defined as root-related environments, whereas TM and LM were classified as leaf-related environments. **(A)** The number of specific and common genes in *S*. Typhimurium 14028s exposed to root-related, leaf-related, or related to both environments. Numbers of upregulated genes in particular environments were presented by the horizontal bars. Connecting lines between single environments (represented by dots) indicate overlapping genes, the number of which was presented above in the corresponding position. **(B)** Enriched biological processes in GO terms of root- and leaf-related conditions. The GO terms were identified using GENEONTOLOGY (http://geneontology.org/), with “*Salmonella* Typhimurium” chosen as the target organism. PANTHER overrepresentation test with Fisher's exact test corrected by calculating the false discovery rate (FDR <0.05) was executed. Genes that were included in both defined niche-related environments **(A)** and within corresponding enriched GO terms **(B)** were identified as candidates for further verification.

To limit the number of potential candidate genes, four steps were undertaken. First, DS soil, TE, and LE were defined as root-related environments, whereas TM and LM were classified as leaf-related environments. Second, commonly upregulated genes in *S*. Typhimurium 14028s identified from root-related environments and leaf-related environments were determined: 47 genes in root-related environments, 29 genes in leaf-related environments, and 48 genes in environments related to both (Figure 2A). Third, enriched biological processes of the defined environments were identified (Figure 2B), such as, translation and carboxylic acid metabolic process in root-related environments, as well as response to heat, arginine biosynthetic process, glycolytic process, and tRNA aminoacylation for protein translation in leaf-related environments. Fourth, genes obtained in step 2, which could also be mapped to the enriched biological processes, were selected as potential candidates. As a result, 3-oxoacyl-[acyl-carrier-protein] reductase encoding gene *fabG*, functional in fatty acid biosynthesis, and phosphoenolpyruvate synthase encoding gene *pps*, were chosen as root-related gene candidates. Leaf-related gene candidates included chaperone protein DnaJ encoding gene *dnaJ*, ATP-binding subunit of serine protease encoding gene *clpA*, pyruvate dehydrogenase subunit E1 encoding gene *aceE*, acetyltransferase component of pyruvate dehydrogenase complex encoding gene *aceF*, and phosphofructokinase encoding gene *pfkB*. As for gene candidates related to both ecological niches, we identified threonine-tRNA ligase encoding gene *thrS*, enolase encoding gene *eno*, fructose-bisphosphate aldolase encoding gene *fba*, and glyceraldehyde-3-phosphate dehydrogenase*-*encoding gene *gapA*. ClpA and DnaJ are chaperone proteins that participate in either ATP-binding or a range of other cellular processes. Threonine-tRNA ligase ThrS is essential for RNA modification. PfkB, Fba, GapA, and Eno are responsible for glycolysis, while AceE and AceF are vital subunits in the pyruvate oxidation process (Table 1).

**Table 1:** Genes contributing to *S*. Typhimurium 14028s adaptation to root-related, leaf-related, or related to both environments.

**Environment**	**Gene**	**Protein function**
Root	*fabG*	3-oxoacyl-[acyl-carrier-protein] reductase
	*pps*	Phosphoenolpyruvate synthase
Leaf	*aceE*	Pyruvate dehydrogenase E1 component
	*aceF*	Acetyltransferase component of pyruvate dehydrogenase complex
	*dnaJ*	Chaperone protein DnaJ
	*pfkB*	Phosphofructokinase
	*clpA*	ATP-binding subunit of serine protease
Both	*eno*	Enolase
	*fba*	Fructose-bisphosphate aldolase
	*gapA*	Glyceraldehyde-3-phosphate dehydrogenase
	*thrS*	Threonine–tRNA ligase

**Figure 3:**
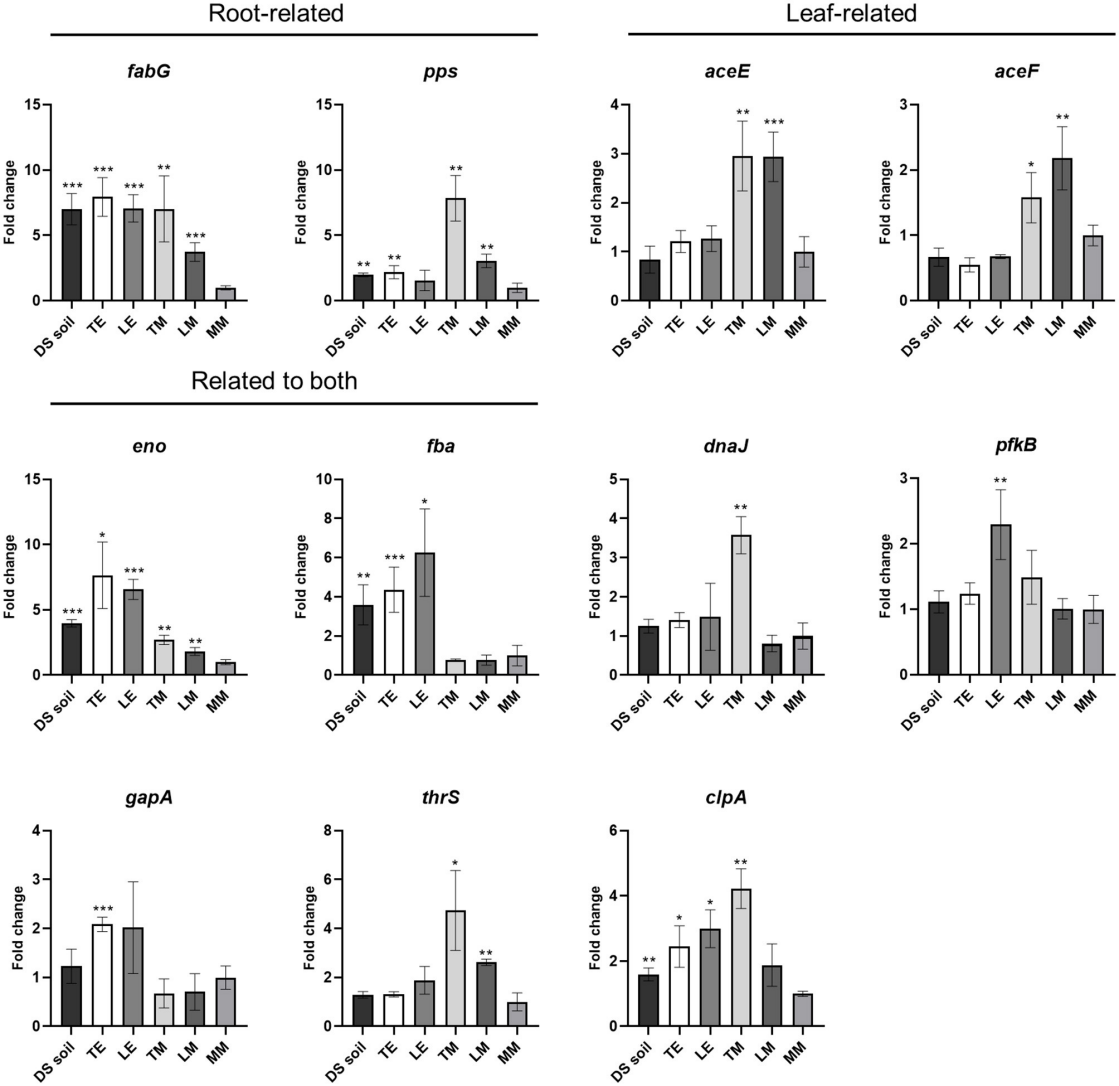
Candidate gene expression in *S*. Typhimurium 14028s incubated in different environments. Gene expression in *S*. Typhimurium 14028s cells recovered from DS soil suspension, TE, LE, TM, and LM was evaluated. Data were normalized to the calibrator *rfaH*. Student's *t*-test was used to compare the gene expression of cells grown in agricultural environment-based media to that of cells grown in MM (control). The error bars reflected the standard deviation among four biological replicates. ^*^p < 0.05; ^**^p < 0.01; ^***^p < 0.001.

To further validate the expression of the putative niche-related genes, an RT-qPCR approach was used. After 24 h incubation of *S*. Typhimurium 14028s in DS soil suspension, TE, LE, TM, and LM, gene expression levels in *Salmonella* cells grown in each medium were compared to those in bacterial cells incubated in MM. Of the five candidates chosen as leaf-related, *aceE* and *aceF* showed expression patterns consistent with RNA-Seq results and an upregulation in TM and LM media. The expression of *eno* demonstrated consistent upregulation in all environments (Figure 3). Given the consistency in results and the importance of *Salmonella* contamination in leafy greens, in the next steps, we focused the study on leaf-related environments.

### Opposite persistence abilities of the *aceE* and *aceB* mutants in tomato leaves

Compared to the soil environment, *Salmonella* contamination of plant leaves poses a direct public health risk, which increases with the popularity of raw-consumed salad (EFSA, 2022a). Although tomato fruit is consumed, earlier research has shown that *Salmonella* can translocate from contaminated leaves to uncontaminated parts, including fruits (Gu et al., 2011; Zarkani et al., 2019). Consequently, tomato leaves were used for further studies.

On the one hand, the upregulation of *aceE* and *aceF*, confirmed by RNA-Seq and RT-qPCR, suggested an important role in the pyruvate dehydrogenase complex for *S*. Typhimurium 14028s growth in TM. We expected, therefore, that a disruption of the complex function by the mutation of *aceE*, which is located upstream of *aceF*, would affect *Salmonella*'s persistence ability. On the other hand, we speculated whether the glyoxylate pathway, which has been linked to *Salmonella*–tomato root interactions (Zarkani et al., 2019), would be involved in tomato leaf persistence as well. In the glyoxylate pathway, malate synthase A (encoded by *aceB*) catabolizes glyoxylate into malate.

**Figure 4:**
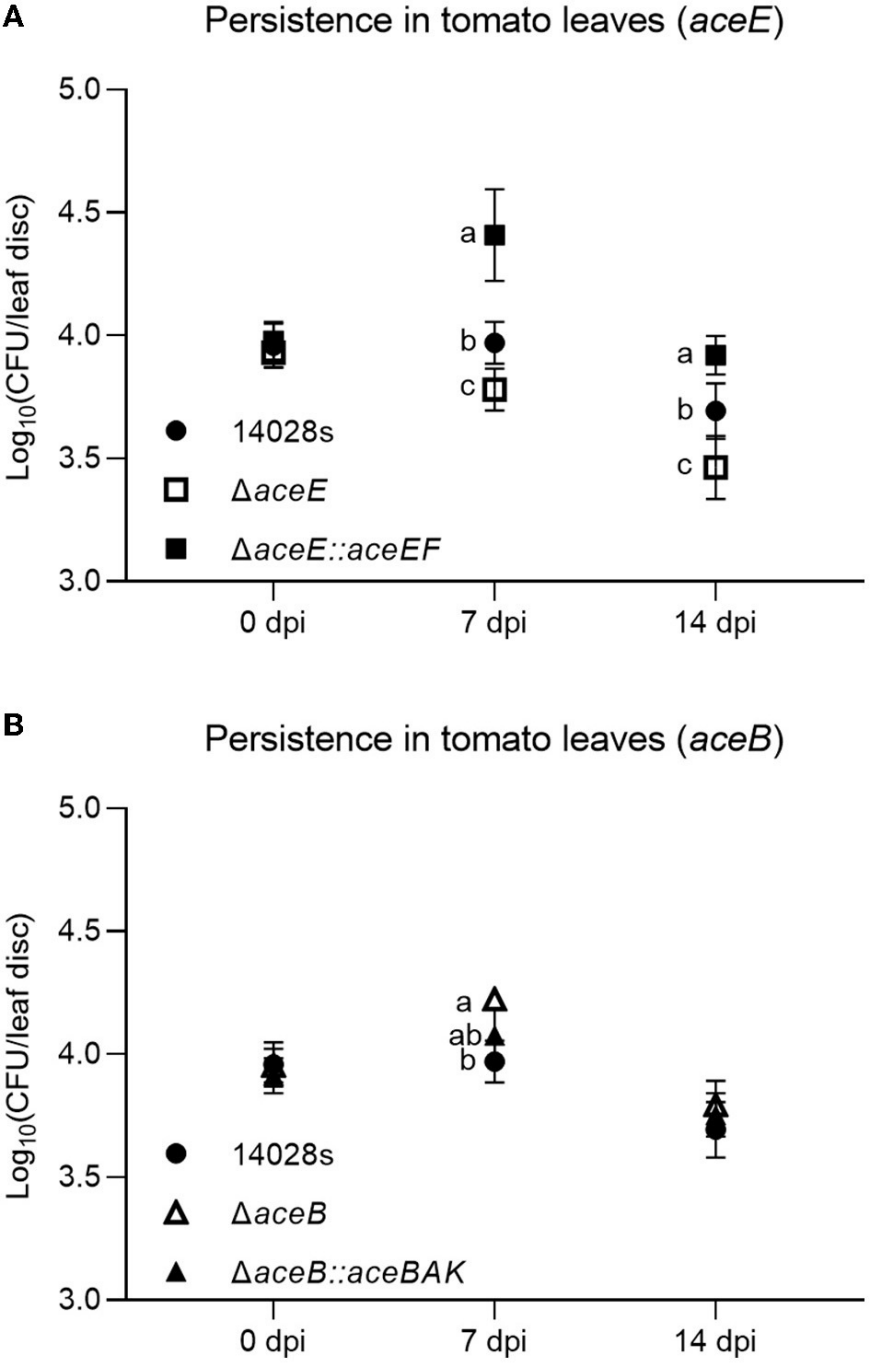
Persistence of *S*. Typhimurium 14028s strains mutated and complemented in *aceE* and *aceB* in tomato leaves. Persistence of *S*. Typhimurium 14028s, mutants, and complemented strains in tomato leaves was evaluated at 0 (3 hpi), 7, and 14 dpi. Single strain suspension at a concentration of 10^7^ CFU/mL was infiltrated into tomato leaves. Persistence was evaluated by the log_10_ CFU number of each leaf disk. **(A)** Persistence of *S*. Typhimurium 14028s, Δ*aceE*, and Δ*aceE*::*EF*. **(B)** Persistence of *S*. Typhimurium 14028s, Δ*aceB*, and Δ*aceB*::*aceBAK*. One-way ANOVA with the Tukey HSD test was used to verify the difference in the wild type, mutant, and corresponding complemented strain at each time point. The error bars indicate the standard deviation among the four biological replicates. The significant difference (*p* < 0.05) was indicated by different letters.

To verify those assumptions, *S*. Typhimurium 14028s, *aceE* and *aceB* mutant strains (Δ*aceE* and Δ*aceB*), and genetically complemented strains (Δ*aceE*::*aceEF* and Δ*aceB*::*aceBAK*) were used. Gene expression of *aceE* or *aceB* in the complemented strains was similar to the level in *S*. Typhimurium 14028s cultured in TM, while no signal was detected in the corresponding mutant strains (Supplementary Figure S3). For further verification *in vivo*, a bacterial suspension was infiltrated into tomato leaves. Compared to *S*. Typhimurium 14028s, Δ*aceE* showed deficient persistence both 7 and 14 days post-inoculation (dpi), while complementation recovered that deficiency (Figure 4A). On the contrary, Δ*aceB* displayed enhanced persistence abilities. At 7 dpi, Δ*aceB* was present in a higher CFU number than *S*. Typhimurium 14028s, while no difference in persistence was observed between Δ*aceB*::*aceBAK* and *S*. Typhimurium 14028s (Figure 4B). This phenomenon may indicate probable contradictory adaptation strategies employed by both mutants during tomato leaf persistence.

### Fumarate is a key metabolite contributing to *Salmonella* persistence in tomato leaves

**Figure 5:**
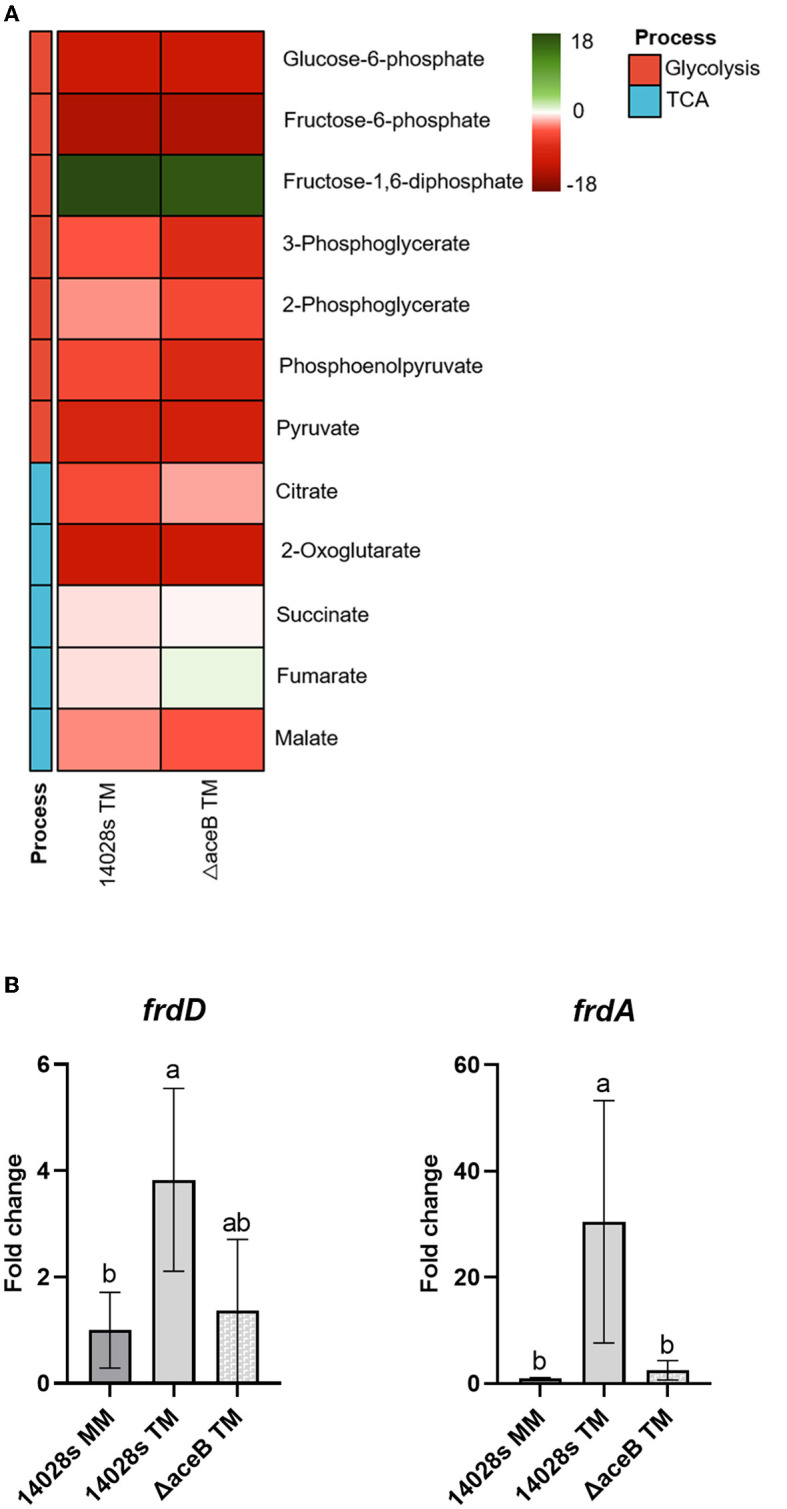
Change in central carbon metabolites and expression of fumarate catabolism genes in *aceB* mutant. Samples of *S*. Typhimurium 14028s and Δ*aceB* incubated in TM were harvested for both metabolite measurement and gene expression evaluation. **(A)** Relative metabolite abundances in the heatmap are displayed as log_2_ fold changes of *S*. Typhimurium 14028s and Δ*aceB* in TM compared to MM. **(B)** Expression of fumarate reductase genes (*frdD* and *frdA*) in *S*. Typhimurium 14028s and Δ*aceB*. Data were normalized to the calibrator *rfaH*. One-way ANOVA with the Tukey HSD test was used for difference evaluation. The error bars indicate the standard deviation among four biological replicates. The significant difference (*p* < 0.05) was indicated by different letters.

Even though the expression of *aceB* was upregulated in *S*. Typhimurium 14028s grown in TM compared to MM (Supplementary Figure S4A), Δ*aceB* exhibited enhanced persistence ability in tomato leaves. We speculated that the mutation of *aceB* induced a compensatory or redundant process or metabolite flux, which assisted in Δ*aceB* persistence in tomato leaves. Since *aceB* plays a major role in carbon metabolism, the abundances of main metabolites in glycolysis and the TCA cycle of *S*. Typhimurium 14028s and Δ*aceB* incubated in TM were evaluated by GC-MS. In comparison to the control (*S*. Typhimurium 14028s grown in MM), abundances of multiple metabolites were altered, albeit to variable degrees (Figure 5A), indicating a metabolism adjustment of both strains to the TM environment. Notably, fumarate was the sole metabolite that reversed the changing direction, from a decreased abundance in *S*. Typhimurium 14028s to an increased abundance in Δ*aceB*. Moreover, the fumarate abundance in the mutant in *pyruvate dehydrogenase subunit E1* (Δ*aceE*), which had lower persistence ability in tomato leaves, was significantly lower than that in *S*. Typhimurium 14028s when grown in TM (Supplementary Figure S5). We hypothesized, therefore, that fumarate abundance in *Salmonella* may affect, to some extent, its persistence in leaves.

Fumarate can be accumulated through upregulated biosynthesis or economical catabolism. In the catabolic pathway, fumarate can be catabolized by fumarate reductase (*frdDCBA*) to succinate. To investigate the possible origin of the fumarate accumulated in Δ*aceB*, we evaluated the expression of *frdD* and *frdA* in Δ*aceB* and *S*. Typhimurium 14028s using RT-qPCR. Compared to the control (*S*. Typhimurium 14028s grown in MM), *frdD* and *frdA* expressions in *S*. Typhimurium 14028s were upregulated in TM (Figure 5B). However, expressions of *frdD* and *frdA* in Δ*aceB* decreased in TM compared to *S*. Typhimurium 14028s (Figure 5B). These results implied a possible accumulation of fumarate in Δ*aceB* grown in TM because of a diminished catabolism ratio.

## Discussion

Agricultural environments serve as a reservoir for *Salmonella* as well as a vector for its transmission toward humans (Schierstaedt et al., 2019). Bulk soil, including DS soil, appears to be an environment with populations of competing microbial communities (Schreiter et al., 2014; Schierstaedt et al., 2020). However, unlike in particular mammals, plant surface-attached or internalized *Salmonella* causes few symptoms in plants (Johnson et al., 2020; Montano et al., 2020). As a result, in such agricultural environments, *Salmonella* may opt to employ rather fundamental means to persist, such as the adaptation of its carbon metabolism, rather than specialized pathogen–host mechanisms. The availability of carbon sources is an important factor affecting bacterial carbon metabolism. Our results indicated that glucose and fructose are abundantly available in tomato and lettuce leaf-based media. Those two sugars were also universally detected in the leaves of plants including cabbage (*Brassica oleracea*), spinach (*Spinacia oleracea*), mugwort (*Artemisia*), green tea (*Camellia sinensis*), *Phalaenopsis*, and rubber tree (*Hevea brasiliensis*) (Kataoka et al., 2004; Shanmugavelan et al., 2013; Zhu et al., 2018). Differently from leaves, glycerol was the predominant carbon source in DS soil; however, the source still needs to be elucidated. In tomato or lettuce root exudates, glucose, fructose, and glycerol were detected (Kamilova et al., 2006; Neumann G. et al., 2014), indicating that the rhizosphere may provide diverse carbon sources. Notably, although organic acids including succinate, fumarate, and malate were detected in tomato and lettuce leaf-based media (Figure 1A) as well as root exudates (Kamilova et al., 2006; Neumann G. et al., 2014), citrate was detected only in TM (Figure 1A) and tomato root exudates (Kamilova et al., 2006), not in LM (Figure 1A) or lettuce root exudates (Neumann G. et al., 2014), underlining the plant specificity.

The impacts of altered microbial carbon metabolism on the colonization of respective hosts or ecological niches have been widely elucidated (Sit et al., 2015; Cole et al., 2017; Prusky and Wilson, 2018; Zhang et al., 2018; Hudson et al., 2022). In the case of *S. enterica*, its persistence in agricultural environments, such as soil, tomato, lettuce, or cilantro (*Coriandrum sativum*), seemed related to carbon metabolism as well (Goudeau et al., 2013; de Moraes et al., 2017; Jechalke et al., 2019; Zarkani et al., 2019; Schierstaedt et al., 2020). Even though the precise source of carbon available for *Salmonella* in different plant tissues remains to be elucidated, some scenarios seem probable. Phyllosphere, rhizosphere, apoplasts, xylem, phloem, and cells represent ecological niches in which nutrients are relatively abundant and might be provided to associated microorganisms, including pathogens (Fatima and Senthil-Kumar, 2015). As discussed above, those niches have different nutrient availabilities. On the one hand, sugars are mainly present in phloem and leaf apoplastic fluid (Fatima and Senthil-Kumar, 2015), where organic acids are also abundant (Lopez-Bucio et al., 2000). Colonization of leaf apoplasts by *Salmonella* has been reported on several occasions (Warriner et al., 2003; Kroupitski et al., 2009a; Chalupowicz et al., 2021; Dixon et al., 2022); therefore, sugars and organic acids present in leaf apoplastic fluid would be accessible. On the other hand, cell wall degrading enzymes (CWDEs) contribute to the invasion of the plant host. Carboxymethylcellulose, which has high homology to CWDEs, can be produced by *Salmonella* and might contribute to the degradation of cell walls (Fratty et al., 2022) and therefore nutrient acquisition. In addition, manual processing, for example, chopping of the leafy greens, can lead to nutrient leakage and may influence *Salmonella* adaptation. Multiple *Salmonella* serovars were found to be attached preferentially to cut or shredded plant materials (Chang and Fang, 2007; Kroupitski et al., 2009b; Patel and Sharma, 2010). *Salmonella* can also benefit from co-infection with plant pathogens, such as *Xanthomonas campestris* pv. *vesicatoria and Pseudomonas syringae* pv. *toma*to (Barak and Liang, 2008; Meng et al., 2013), which would further improve nutrient availability.

Glucose is the main carbon source and is catabolized through glycolysis in *Salmonella*. As alternatives, PPP or KDPGP might be activated through glucose-6-phosphate, which is produced in the initial step of glycolysis. These two latter pathways are commonly found in *Salmonella* present in animal cells. In human HeLa and Caco-2 cells, as well as murine J774-A.1 cells, either PPP or KDPGP are required, as suggested by isotope tracer and transcriptome analysis (Eriksson et al., 2003; Hautefort et al., 2008; Gotz et al., 2010). Accordingly, the mutant in glucose-6-phosphate 1-dehydrogenase (Δ*zwf* ) affecting PPP and KDPGP, displayed impaired virulence *in vivo* when using the mice model (Lundberg et al., 1999). In this study, the detection of glucose-6-phosphate but not fructose-6-phosphate in *S*. Typhimurium 14028s cells grown in TM and LM indicated the possibility of a bypass through PPP or KDPGP. This phenomenon could be used to degrade phosphorylated intermediates in bacterial cells, since the accumulation of them, for example, glucose-6-phosphate, could be toxic (Boulanger et al., 2021, 2022).

To some extent, the genes regulated by *Salmonella* in plant-related ecological niches below and above the ground are distinct. Genes related to sugar or fatty acid biosynthesis are predominant in root-related environments (Table 1). In such conditions, nutrients are rarer than in above-ground tissues with functional photosynthesis (Figure 1A). The pyruvate dehydrogenase subunit-encoding gene *aceE* was identified as essential for *Salmonella* growth in TM and survival in tomato leaves (Table 1, Figures 3, 4A). Similarly, *S*. Typhi BRD948 upregulated the expression of *aceE* in yet another important agricultural environment, water (Kingsley et al., 2018). In *S*. Enteritidis, the *aceE* mutant was attenuated in the invasion of epithelial cells and survival in chicken macrophages (Chang et al., 2008; Pang et al., 2011). The deficiency of invasion or colonization in cells or animal hosts was speculated to be caused by the impaired growth of the *aceE* mutant (Pang et al., 2011). However, since the persistence of *S*. Typhimurium 14028s in tomato leaves is probably not based on proliferation (Zarkani et al., 2019, 2020), the diminished persistence of the Δ*aceE* may indeed be caused by the lower adaptation ability.

In contrast to Δ*aceE*'s decreased persistence, mutants in malate synthase A encoding gene *aceB*, functioning in the glyoxylate pathway, displayed better persistence capability in tomato leaves (Figure 4B). In the glyoxylate pathway, AceA functions as isocitrate lyase in the isocitrate shunt to glyoxylate and succinate, upstream of AceB. The glyoxylate pathway has been reported to contribute to pathogen virulence and colonization (Lorenz and Fink, 2001; Dunn et al., 2009). However, there are also some contradictions under certain conditions, for example, *Yersinia pestis* does not require *aceA* during flea infection or mouth pathogenicity (Sebbane et al., 2004). In *Salmonella*, although *aceA* is required for persistence in mice, it is dispensable in acute lethal infection (Fang et al., 2005). In this study, both *aceA* and *aceB* were upregulated in *S*. Typhimurium 14028s grown in TM (Supplementary Figure S4), compared to MM. In addition, 2-oxoglutarate, the product of an isocitrate-sourced shunt in the TCA cycle, was not detectable in *S*. Typhimurium 14028s grown in TM (Figure 1B). These results indicated that the glyoxylate pathway might be involved in *Salmonella* growth in TM. Even though a mutation in either *aceA* or *aceB* downregulated the expression of the other gene (Supplementary Figure S4), it had no effect on *Salmonella* persistence in tomato leaves (Figure 4B and Supplementary Figure S6). Δ*aceB* displayed even better persistence capability, and this predominance disappeared when being genetically complemented (Figure 4B). It seemed that some complimentary pathway was triggered or enhanced, especially in Δ*aceB*, when the glyoxylate pathway of *Salmonella* was impaired during adaptation to tomato leaves.

**Figure 6:**
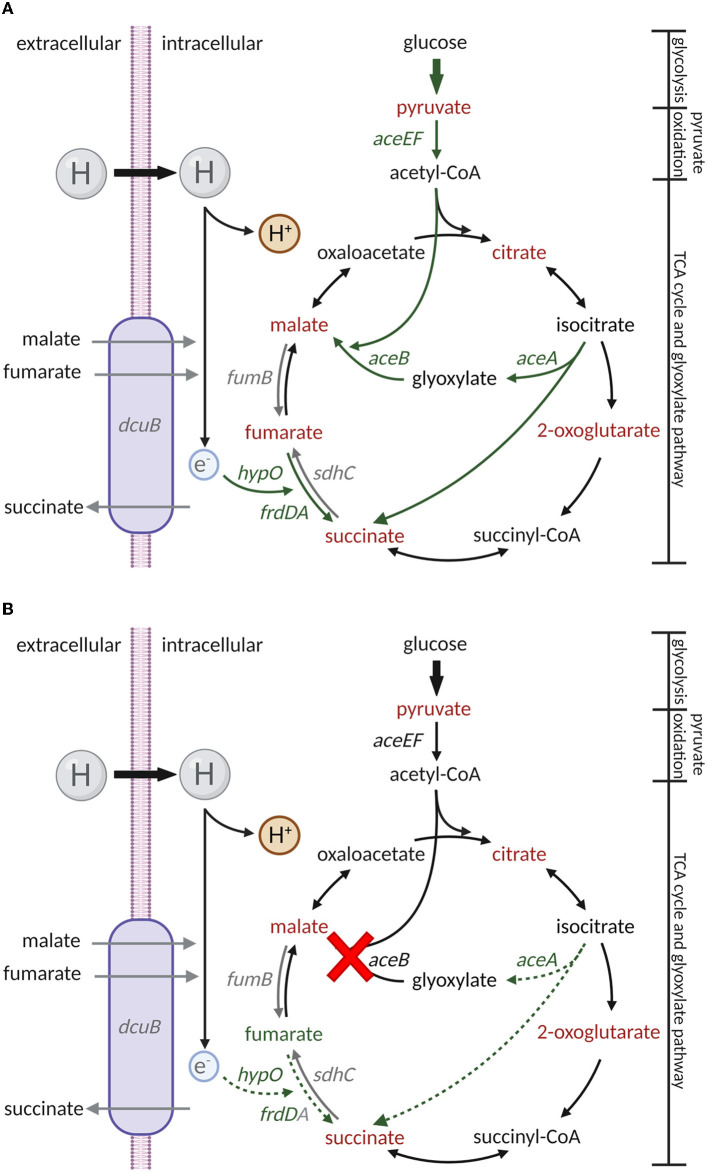
Putative regulatory processes in carbon metabolism of *S*. Typhimurium 14028s and *aceB* mutant in TM. Regulatory processes in carbon metabolism in *S*. Typhimurium 14028s **(A)** and Δ*aceB* **(B)** in TM were displayed based on the results from metabolite abundance and gene expression analysis. Arrows with bold solid lines, solid lines, and dot lines represent abbreviated processes, metabolism/regulatory direction, and impaired processes, respectively. Red indicates a decrease, green indicates an increase, and gray indicates no changes. Created with BioRender.com (Publication License: TJ25NTL8BO and TO25NTKYMA).

While analyzing the metabolite abundance, fumarate accumulation was identified as one of the potential reasons for enhanced Δ*aceB* persistence in leaves (Figure 5A). The biosynthesis and catabolism of fumarate are multidirectional, depending on the processes in which it is involved. Aside from its role in the TCA cycle, fumarate serves as an electron acceptor (Tomasiak et al., 2007). Fumarate dehydrogenase-encoding genes *frdA* and *frdD* (Figure 5B), rather than succinate dehydrogenase-encoding gene *sdhC* (Supplementary Figure S7A) in the TCA, were upregulated in the TM using both RNA-Seq (Zarkani et al., 2019) and RT-qPCR (Figure 5B). A hypothetic regulatory pathway of *S*. Typhimurium 14028s (Figure 6A) and Δ*aceB* (Figure 6B) in TM was shown. Fumarate catabolism by fumarate dehydrogenase might be reduced in Δ*aceB*, resulting in fumarate accumulation. In addition, the expression of the hydrogen transporter (*hypO*), acting as an electron donor, was also reduced compared to *S*. Typhimurium 14028s, although not significantly (Supplementary Figure S7B). Interestingly, gene expression of the fumarase B (*fumB*) or C4-dicarboxylate transporter (*dcuB*) in both *S*. Typhimurium 14028s and Δ*aceB* grown in TM was comparable to that grown in MM (Supplementary Figures S7C, S7D), which could be the reason why, even if malate and fumarate are abundant in TM (Figure 1A), we observed no accumulation of fumarate in *S*. Typhimurium 14028s. However, the association between the *aceB* mutation and diminished fumarate catabolism still needs to be investigated in the future.

Taken together, we described a general pattern of adaptation to carbon sources used by the human pathogen *Salmonella enterica* in agricultural environments. We provided candidate genes regulated in particular ecological niches, which could be a premise for future mechanistic studies. As an example of leaf environment-related genes, *aceE* and *aceB* were studied in more detail. We postulate that the improved persistence of Δ*aceB* might be caused by the accumulation of fumarate in bacterial cells present in leaves, and the deficient persistence of Δ*aceE* was also fumarate-related. The understanding of the mechanisms used by *Salmonella* to utilize carbon sources could lead to prospective strategies to diminish or perhaps prevent *Salmonella*'s long-term persistence in agricultural environments.

## Data availability statement

The original contributions presented in the study are included in the article/Supplementary material, further inquiries can be directed to the corresponding author.

## Author contributions

MHa, JS, SJ, and AS conceived the research project and contributed to the experimental design. Experimental study was performed by MHa with the assistance of YD. MN and MHe provided bacterial mutants and complemented strains. JW and MN-S executed GC-MS and raw data analysis. MHa performed data analysis, designed figures, and wrote the first version of the manuscript. All authors contributed to the manuscript's improvement and revision.
