# Supplementary figures and images for: Salmonella enterica relies on carbon metabolism to adapt to agricultural environments
Source: Front Microbiol. 2023 Sep 7;14:1213016. doi: 10.3389/fmicb.2023.1213016 (PMC10513388; doi:10.3389/fmicb.2023.1213016)

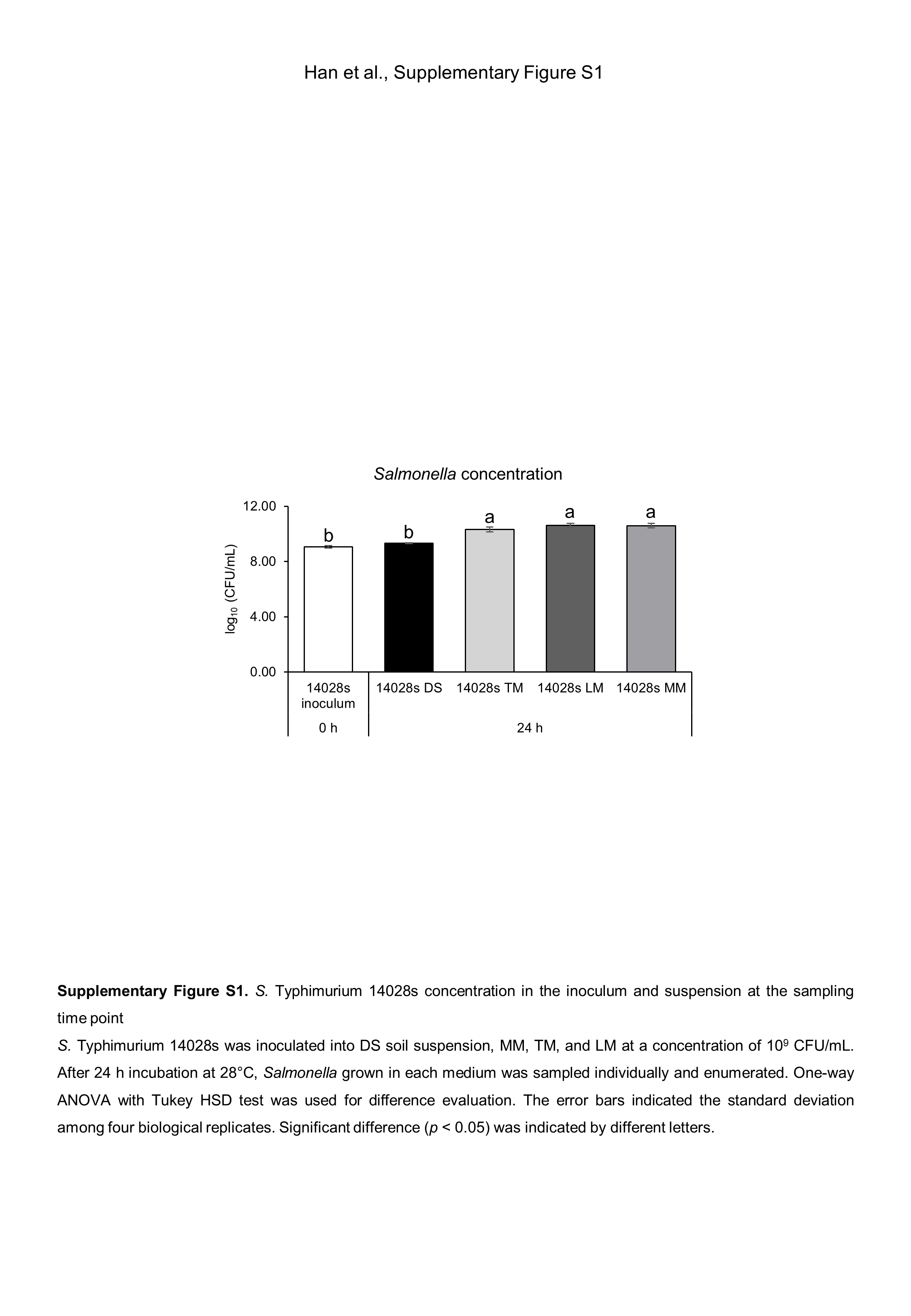

Supplement: Supplementary file 1 [file Image_1.TIF]

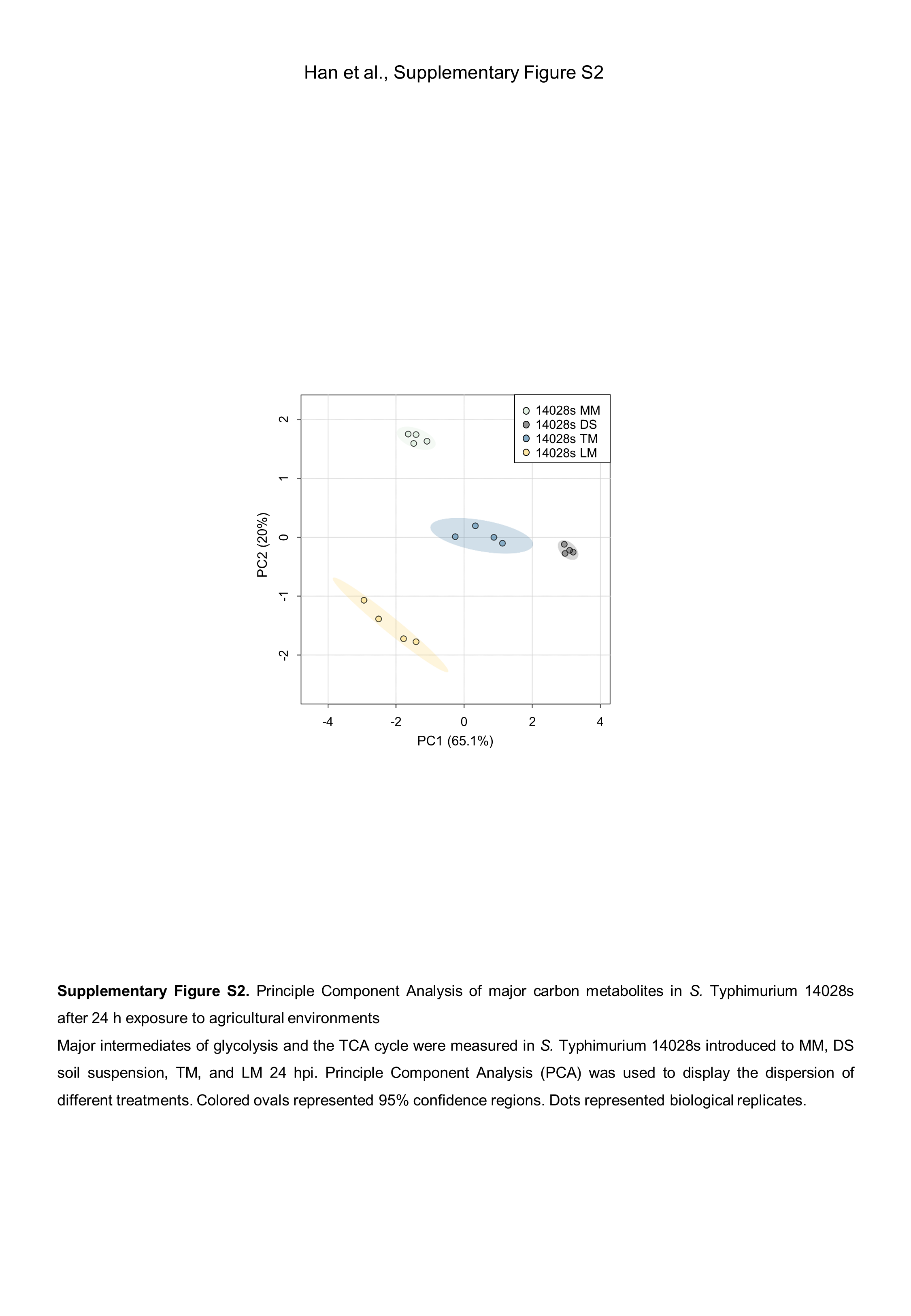

Supplement: Supplementary file 2 [file Image_2.TIF]

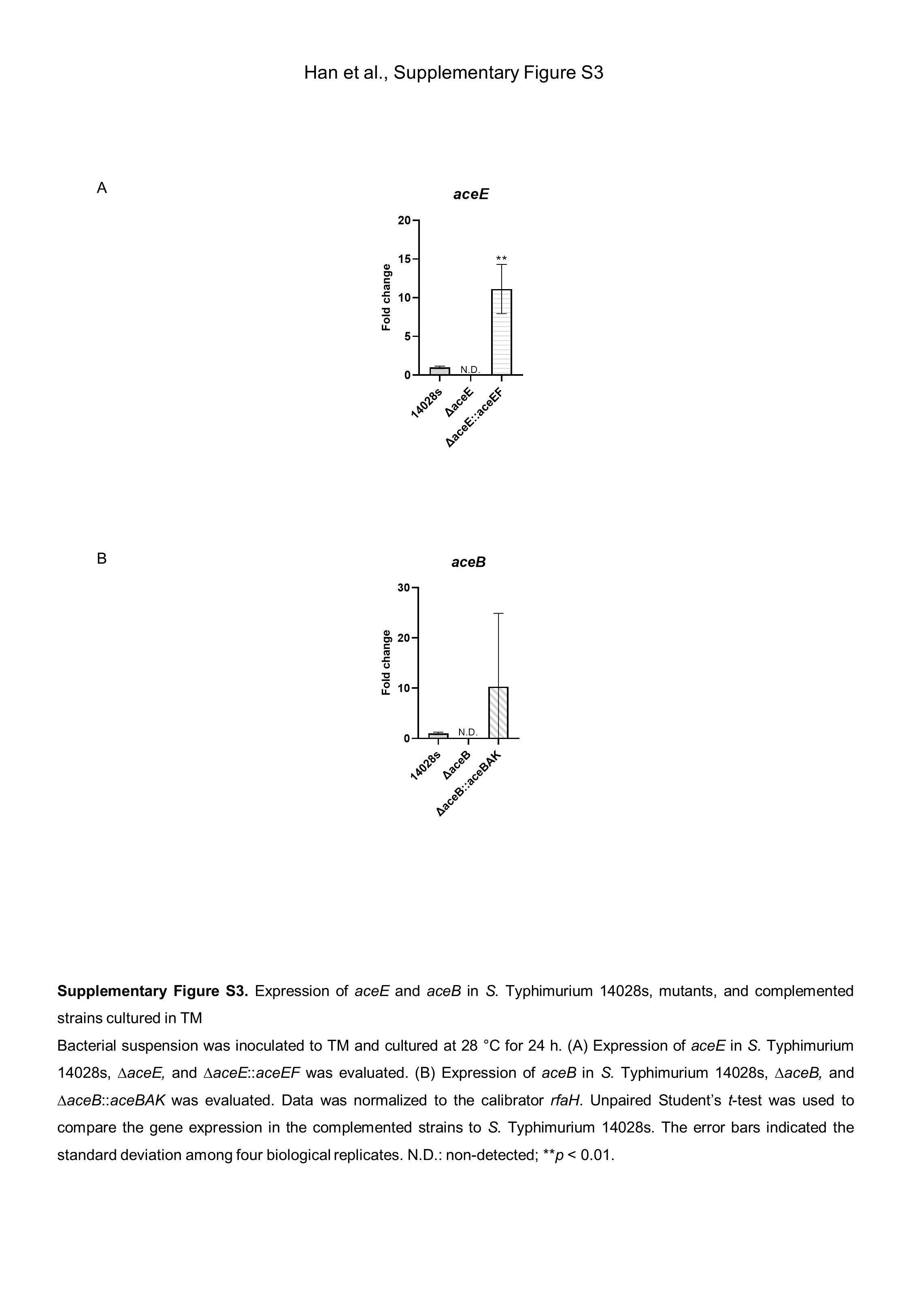

Supplement: Supplementary file 3 [file Image_3.TIF]

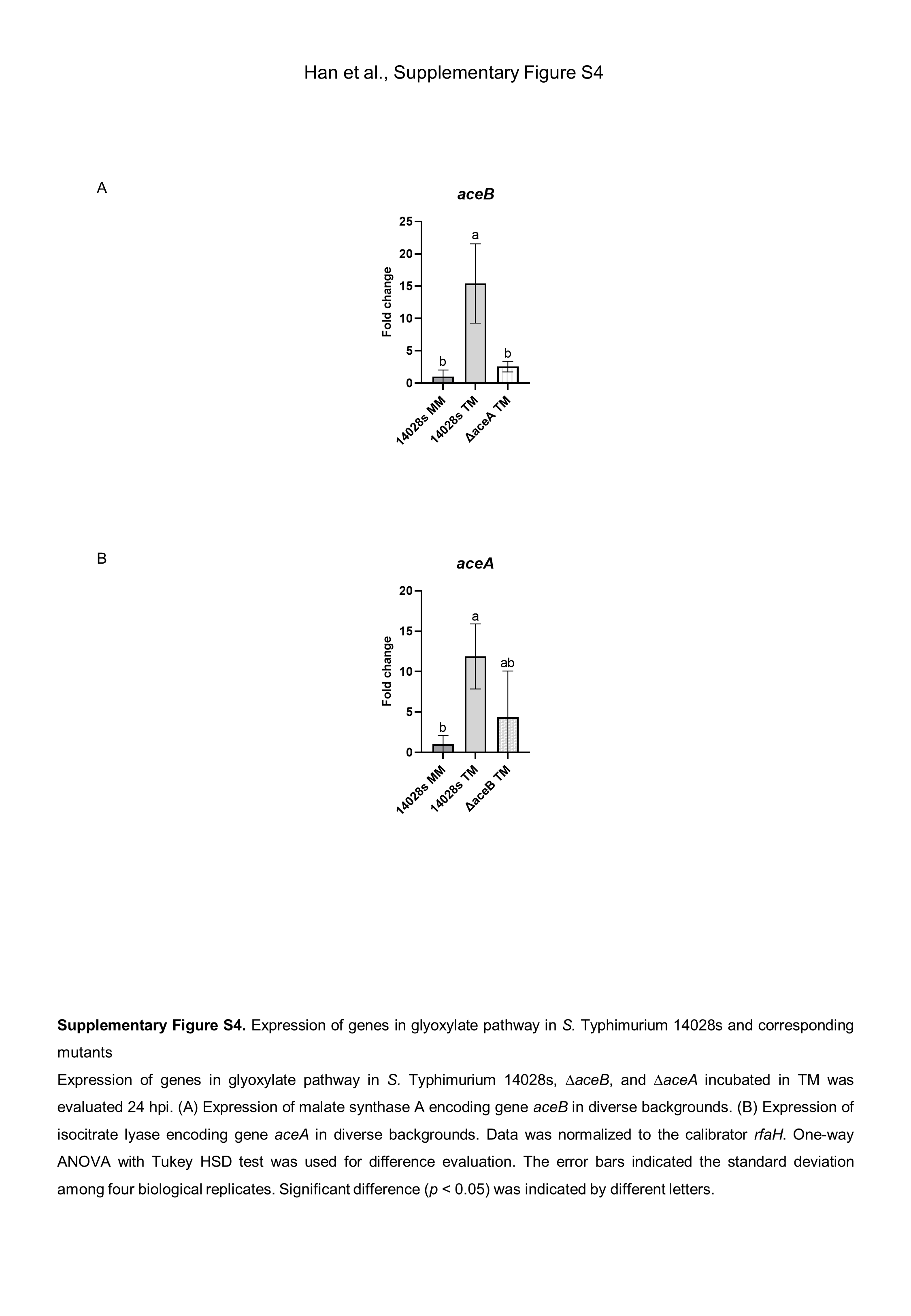

Supplement: Supplementary file 4 [file Image_4.TIF]

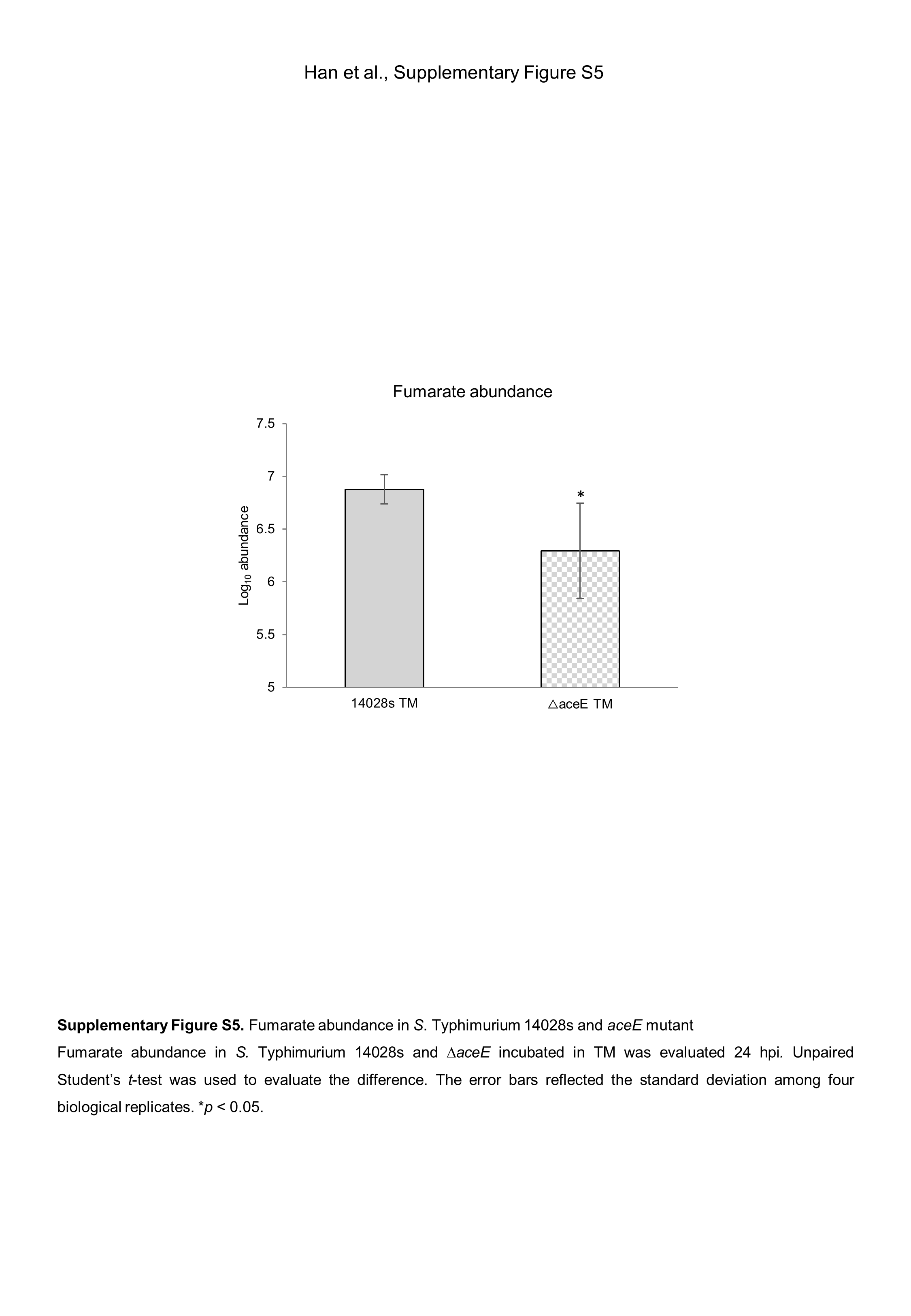

Supplement: Supplementary file 5 [file Image_5.TIF]

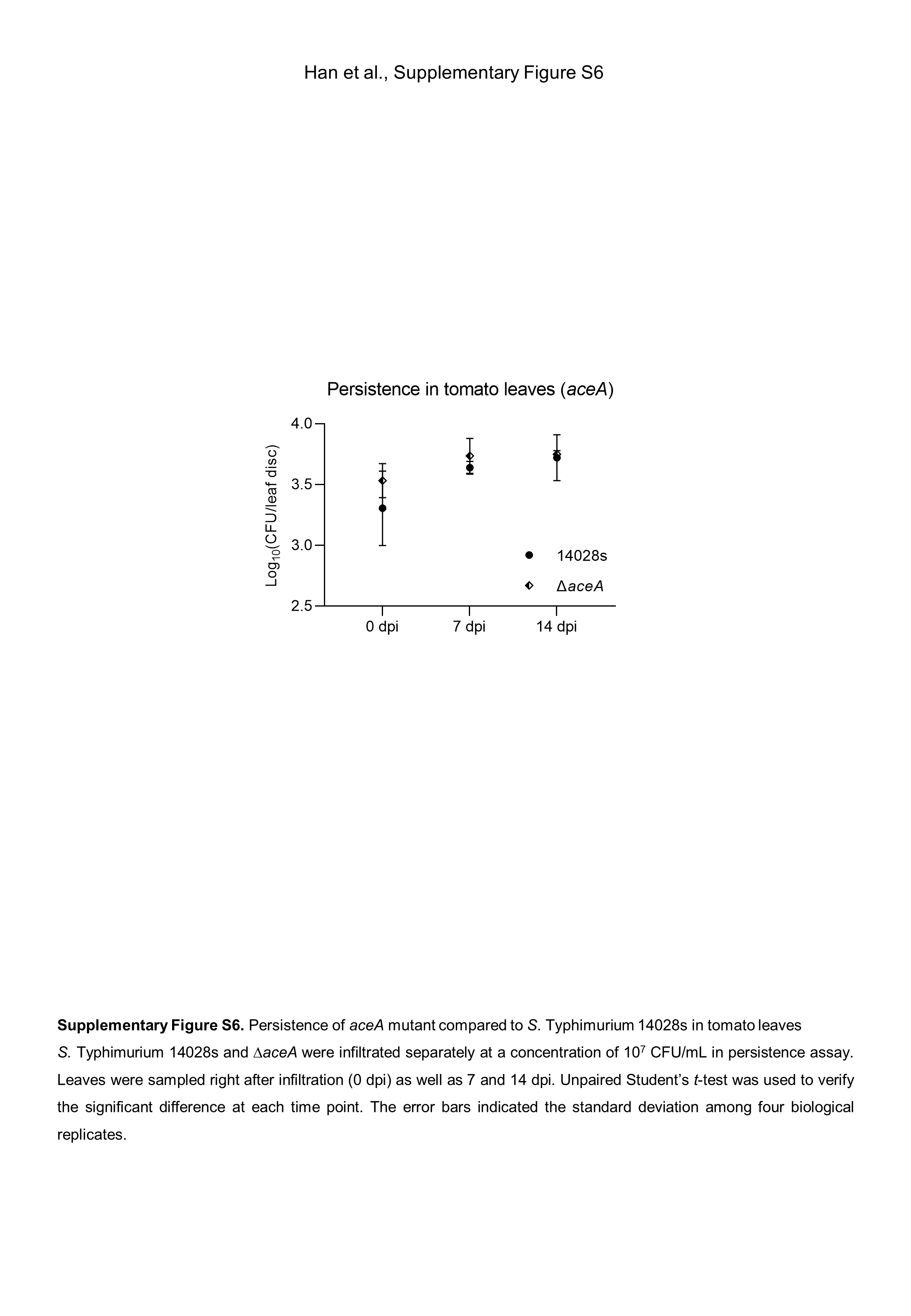

Supplement: Supplementary file 6 [file Image_6.TIF]

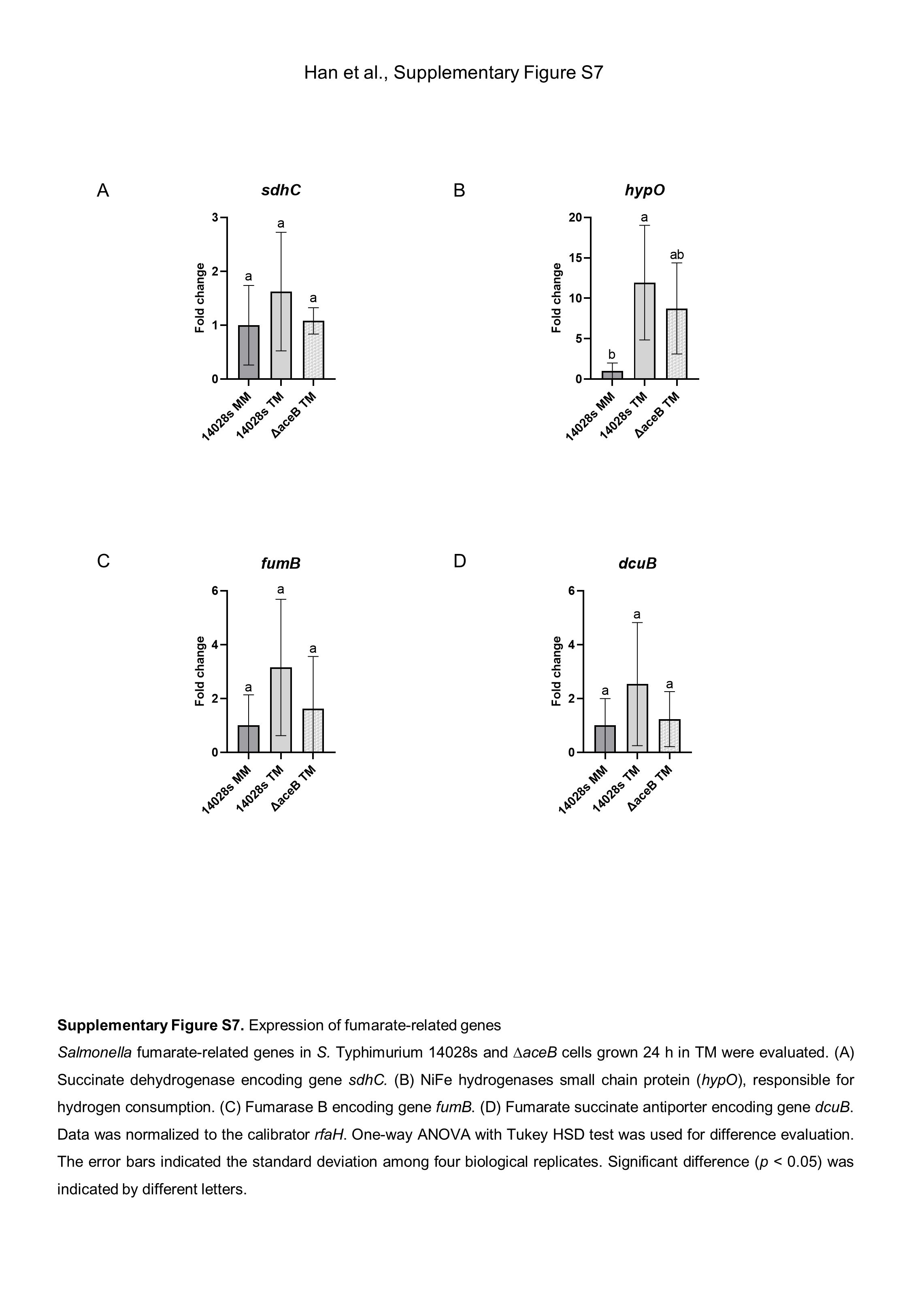

Supplement: Supplementary file 7 [file Image_7.TIF]

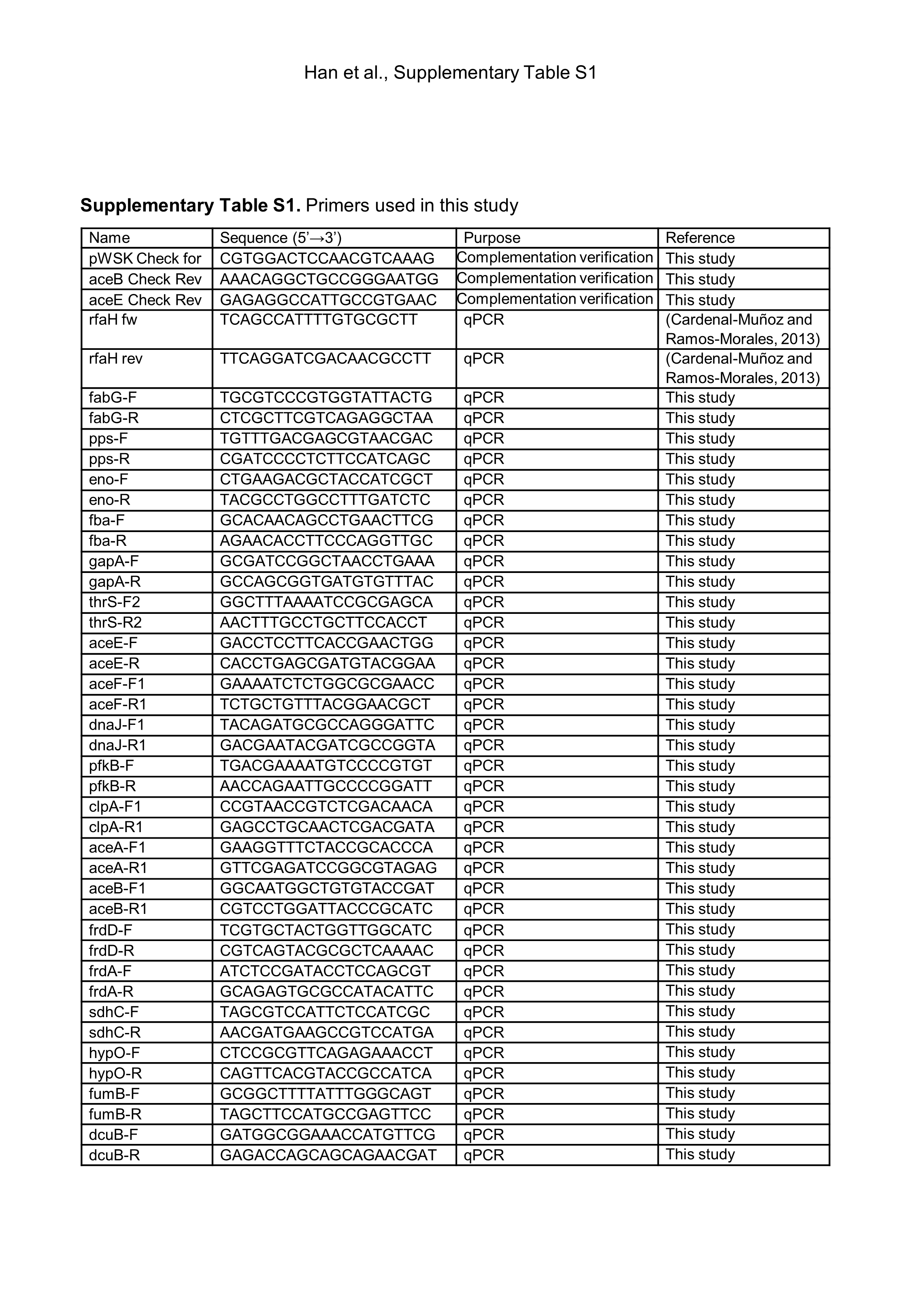

Supplement: Supplementary file 8 [file Image_8.TIF]
